# Maxillary Sinus Inflammatory Myofibroblastic Tumors: A Review and Case Report

**DOI:** 10.1155/2015/953857

**Published:** 2015-02-11

**Authors:** Chase C. Hansen, Colby Eisenbach, Carlos Torres, Suzanne Graham, Fred Hardwicke

**Affiliations:** ^1^Department of Radiation Oncology, Texas Tech University Health Sciences Center, Lubbock, TX 79430, USA; ^2^Department of Pathology, Texas Tech University Health Sciences Center, Lubbock, TX 79430, USA; ^3^Department of Internal Medicine, Texas Tech University Health Sciences Center, Lubbock, TX 79430, USA

## Abstract

An inflammatory myofibroblastic tumor (IMT) is an immunohistochemically diverse entity demonstrating neoplastic and nonneoplastic qualities. Although IMTs can arise in any area of the body, lesions arising in certain sites, namely, the nasal cavity, paranasal sinuses, and pterygopalatine fossa, demonstrate a heightened neoplastic and invasive potential. Despite case specific complete tumor regression and disease remission in response to pharmacotherapeutics, a subset of IMTs remain resistant to all forms of therapy. We present such a case, a 34-year-old female patient, with a highly resistant, maxillary sinus IMT. Her refractory, ALK-1 negative IMT has not responded well to novel therapies reported in current literature. This case suggests the role of zonal expressivity within a single lesion as a probable mechanism for its highly resistant nature and should promote determination of each IMT's cytogenetic profile to provide more effective targeted therapy. Paper includes a literature review of all maxillary sinus IMTs from 1985 to 2014 along with their immunohistochemical staining, treatments, and outcomes.

## 1. Introduction

An inflammatory myofibroblastic tumor (IMT) is an immunohistochemically diverse entity demonstrating neoplastic and nonneoplastic qualities first described by Brunn in 1939 [1, 2]. Until recently, the umbrella term “inflammatory pseudotumor” has been used to describe these lesions, which share a common histological appearance. A variable degree of spindle cell proliferation within a background of myxoid/collagenous stroma and a significant inflammatory infiltrate composed of lymphocytes, histiocytes, plasma cells, neutrophils, and eosinophils is descriptive of such lesions [3, 4].

Although the term inflammatory myofibroblastic tumor was coined by Umiker and Iverson in 1954 [2], the prominent histologic variance, erratic neoplastic, and inflammatory characteristics of these lesions have led to the development of a diverse nomenclature including, but not limited to, fibroxanthoma, plasma cell granuloma, histiocytoma, and inflammatory fibrosarcoma. Differences in cytological makeup and vast variations in the cytogenetic expression of immunohistochemical markers and inflammatory mediators present a challenge to providing accurate diagnosis, a process which has not been determined. Although IMTs can arise in any area of the body, lesions arising in certain sites, namely, the nasal cavity, paranasal sinuses, and pterygopalatine fossa, demonstrate a heightened neoplastic and invasive potential [5–12].

Treatment, in like manner to pathological diagnosis, is often a quandary for surgeons and oncologists alike. To date, complete surgical excision or corticosteroid therapies of IMTs are the gold standards of treatment [13]. However, in refractory, recurring or nonresectable cases, systemic and novel therapies have arisen, including chemotherapy, radiotherapy corticosteroids, NSAIDs, COX inhibitors, and kinase inhibitors [14, 15]. Despite case specific complete tumor regression and disease remission in response to such pharmacotherapeutics, a subset of IMTs remains resistant to all forms of therapy. The present case demonstrates the neoplastic/invasive variant of IMT and tumor resistant behavior, despite the use of standard and novel therapeutic approaches.

## 2. Case Presentation

**Figure 1:**
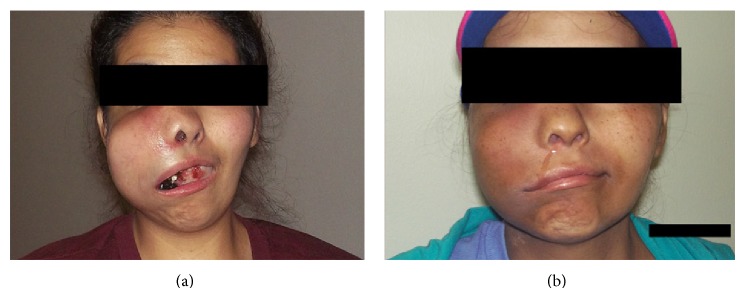
(a) A picture of the patient at initial presentation to our clinic July 2014. A right-sided mass is evident causing trismus and other related mass effect symptoms. The lesion measured 7.1 cm at greatest dimension. (b) A picture of the patient at a 3-month follow-up appointment. Tumor regression as well as accompanying symptomatic relief is apparent when comparing to prior image (a). Lesion measured 4.9 cm at greatest dimension.

**Figure 2:**
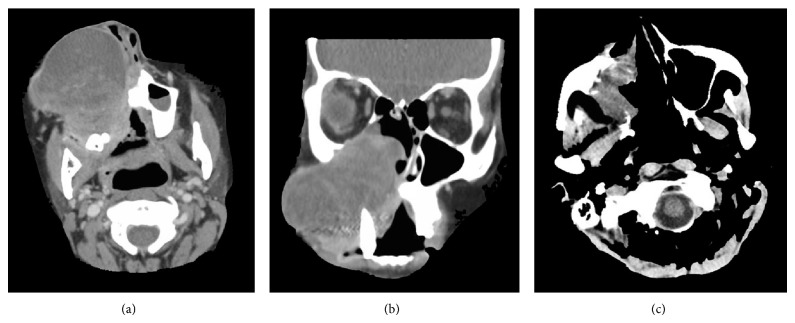
(a) A computed tomographic scan of the maxillofacial region with contrast showing the maxillary IMT in axial section at initial presentation July 2014. (b) A coronal section of the same CT scan highlighting extent of invasion. (c) A CT scan of the head without contract to rule out an intracranial hemorrhage October 2014. Image demonstrates tumor regression when compared with (a) and (b).

A 34-year-old Hispanic female presented with complaints of progressive nasal congestion and mass in the right nasal cavity. Initial biopsy of the mass was felt to be benign. A year later, her right nasal mass recurred and again was reexcised with pathology reported as benign. A few months after her second surgery the mass had rapidly grown in size filling her mouth and causing right facial swelling (Figure 1(a)). She had difficulty eating and swallowing. CT scan of the neck showed an extensive destructive mass measuring 5.5 cm × 5.3 cm × 7.1 cm with extension into the medial wall of maxillary sinus and extending to the inferior orbit (Figures 2(a) and 2(b)). She had evidence of metastases to her right neck but there was no intracranial extension.

The lesion was biopsied and sent to two referral centers for assistance in making the diagnosis. The first referral center determined that the lesion consisted of a “polypoid sinonasal mucosa with ulceration, with acute and chronic inflammation and spindle cell proliferation, favoring reactive changes.” Immunohistochemical stains including smooth muscle antibody (SMA), desmin, S100, and keratin were all negative.

The patient went to second referral center for further treatment. At this institution, the report was slightly different, determining the sample to be consistent with an IMT, but found it had scattered positivity for ALK-1 and SMA within spindled cells. This is significant, because a diagnosis of IMT is quite difficult; however, one would expect samples from the same tumor biopsy to show similar SMA staining. The complexity of this tumor is further noted in that another biopsy, three years following the initial pathology report, differed from both of the previous reads.

**Figure 3:**
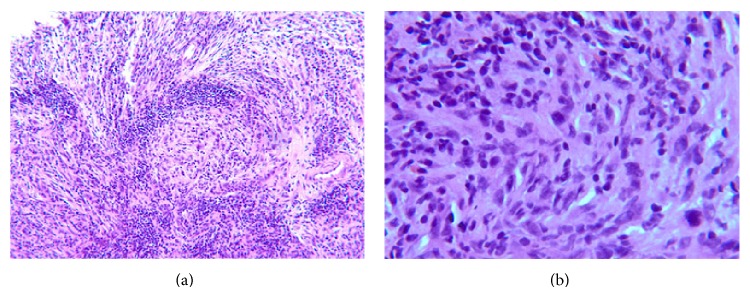
Pathologic findings by hematoxylin and eosin staining of maxillary tumor tissue samples. (a) Whorled and fascicular spindle cells with moderate nuclear atypia and mitotic activity are heavily infiltrated by mixed chronic inflammatory infiltrate at 125x magnification. (b) Similar findings at 500x magnification.

Most recently, the second referral center reported immunohistochemistry stains demonstrating a profile different than the prior biopsies, with rare tumor positivity for SMA, and negativity for S100, pan-keratin, p63, and ALK-1. Next-Generation Sequencing- (NGS-) based analysis for detection of somatic mutations revealed TP53 and KRAS mutations. It was felt that pathology was consistent with inflammatory myofibroblastic tumor (Figures 3(a) and 3(b)). They decided to initiate therapy using crizotinib, an ALK-1 and ROS-1 inhibitor, given the refractory status of the tumor and prior positivity for ALK-1. Given the history, there was a chance this tumor would respond. Unfortunately, this patient did not respond to the targeted therapy and was eventually sent to her hometown hospital to seek further treatment due to unanticipated insurance complications.

In the beginning of July 2014, the patient was seen at our institution. At a multidisciplinary tumor board conference, it was decided that the best courses of action would be combination radiation and chemotherapy. She received a total dose of 60 Gy in 6 weeks using IMRT. She was also treated with ifosfamide, dacarbazine, and mesna as well as celecoxib. She recently completed four cycles of this regimen and a follow-up CT demonstrated overall tumor regression to a measurement of 4.9 cm at its greatest dimension as compared to 7.1 cm at initial presentation (Figures 1(b) and 2(c)).

## 3. Discussion

Since its first known appearance in medical literature in 1939, the inflammatory myofibroblastic tumor has garnered the attention of pathologists, surgeons, and oncologists, alike, due to its marked idiosyncrasies in immunohistology, pathophysiologic behavior, and therapeutic response [13]. In past years, a relative dogma has developed regarding the benign nature of IMT. Many cases have arisen, much like that presented above, which call such notions into question: thus, shedding light on the malignant and aggressive variant of this fascinating neoplasm. Due to the variable, and often case specific, behavior of many IMTs, it is fitting to delve somewhat into the generalities applied to this case and thereafter discuss the unique findings. The ultimate goal is to advance the management and care of individuals affected by IMTs.

The definitive etiology of IMT has yet to be fully elucidated, but a great deal of proponents agree its development is largely multifactorial involving both inflammatory and chromosomal aberrations [16–23]. Undeniably, vast variations in the cytogenetic expression of immunohistochemical markers and inflammatory mediators present a challenge to providing accurate diagnosis and uncovering the concerted symphony that must take place for an IMT to develop.

To date, many cellular markers have been identified, including desmin, vimentin, smooth muscle actin, cytokeratin, and ALK-1 that aid in the pathologic diagnosis of IMT. Of particular interest is anaplastic lymphoma kinase or ALK-1 as well as its essential role in differentiating IMT from other spindle cell neoplasms that fall within the broad category of “inflammatory pseudotumors.” According to studies conducted by Lawrence in 2000 and Coffin in 2001, greater than 50% of soft tissue IMTs possess chromosomal translocations involving the short arm of chromosome 2 and the ALK tyrosine kinase receptor locus [16–19, 24].

The diagnostic value of ALK-1 positivity is evident, considering most of the neoplastic counterparts of IMTs including desmoid fibromatosis, nodular fasciitis, calcifying fibrous tumor, myofibromatosis, and infantile fibrosarcoma are negative for ALK. Despite the obvious identification value of ALK-1 status in these neoplasms, the clinical and prognostic significance remains uncertain with some researchers suggesting unsubstantial value in ALK-1 status. However, adequate evidence exists to demonstrate the role of ALK-1 in conveying metastatic and invasive potential to IMTs. In fact, several recent retrospective studies have shown a marked increase in metastasis and recurrence in IMTs that are ALK-1 negative when compared to ALK-1 positive lesions [4, 25, 26].

In light of such information, ALK-1 negativity alone, as seen in the case presented above, demands an aggressive therapeutic approach and increased vigilance for distant metastases or local recurrence. Additionally, as with any unregulated cell growth resulting in tumor formation, chromosomal aberrations leading to cellular atypia, nuclear pleomorphism, abnormal mitotic rate, DNA aneuploidy, and tumor suppressor inactivation have been shown to be beneficial in predicting IMTs [23] This heightened neoplastic capability gives IMTs potential for particularly aggressive clinical behavior with local recurrence or malignant transformation.

Despite the IMT predilection for developing in the lung, abdominopelvic, retroperitoneal, and extremities in adolescent and pediatric populations, this rare tumor has been shown to occur in a vast age range and in a great number of locations [3, 24]. These include, but are not limited to, the orbit, liver, paranasal sinuses, and bladder [25, 27–30]. Just as certain cell signaling and chromosomal mutations enhance adverse disease behavior, a large degree of case specific evidence, in addition to that found in the case presented above, exists to support the idea that lesions arising in the nasal cavity, paranasal sinuses, and pterygopalatine fossa demonstrate a heightened neoplastic and invasive potential [5–12, 31]. Contiguous spread of such lesions has been shown to result in destruction of surrounding muscles, fat, nerves, and bone. Common presenting symptoms include nasal congestion, swelling, epistaxis, pain, parasthesias, proptosis, and headache [6, 7]. Large lesions, or those arising in areas not amenable to surgical removal, have been shown to have a higher degree of local recurrence and an increased risk for distant metastasis [32].

Of particular interest regarding the etiology of IMT is the role both the immune system and its host of inflammatory mediators play in the development and persistence of these rare entities [19, 20, 33–35]. Historically, IMTs were first described as benign, reactive, postinflammatory lesions arising primarily in children and adolescents [1, 2]. The variable degree of spindle cell proliferation within a background of myxoid/collagenous stroma and a variable inflammatory infiltrate is a testament to such a description.

In the face of such early evidence and the relatively consistent presentation profile within pediatric intrapulmonary lesions, no surprise IMTs have struggled to shed their recurrent inclusion into the misnomer of inflammatory pseudotumor, even in recent years. However, due to the work of Meis and Enzinger in 1990 and Coffin in 1995 involving intra-abdominal and retroperitoneal tumors [3, 28], the invasive and metastatic potential of the newly coined inflammatory myofibroblastic tumor was made evident. Thus, the dichotomy of inflammatory versus neoplastic behavior in IMTs was born. In recent years, the idea that one etiologic mechanism is involved has been largely abandoned and a multifactorial school of thought now predominates.

Just as erratic immunohistochemical characteristics seem to define IMT, differences in the cytological makeup of IMTs have been described and categorized into four cellular variants, largely modified from the three stromal classifications first described by Coffin et al. in 1995 [3, 4]. The fusion of these two classification systems yields the following cellular and stromal combinations:

1. Spindle cells within a vascularized myxoid stroma and an inflammatory infiltrate of neutrophils and eosinophils.
2. Compact spindle cells within a collagenized stroma and storiform architecture and an inflammatory infiltrate of plasma cells and lymphocytes often forming germinal centers.
3. Elongated spindle cells within a hypocellular highly collagenous stroma and a variable inflammatory infiltrate of lymphocytes, plasma cells, and eosinophils.
4. Lymphohistiocytic variant consisting of myofibroblastic spindle cells and foamy histiocytes. This is thought to represent the most inflammatory variant.

With regard to the case in question, it is important to point out the fact that significant overlap in cellular populations can occur and the phenomenon of maturation and zonation within a single tumor has been described: thus, adding to the complexity of affective histological classification and ultimate diagnosis [4]. Of interest, the occurrence of zonal expressivity and variable cellular differentiation within a single tumor has been shown to promote the need for multiple perioperative biopsies or complete sample procurement.

As for the case at hand, two prominent institutions obtained differing pathology reports and scattered reactivity of the previously discussed immunohistochemical markers within the same tumor sample before being seen at our institution. The occurrence of zonation and maturation explains this discrepancy. Additionally, and perhaps most importantly, the awareness of such a phenomenon within IMTs is paramount to successful diagnosis and treatment of inflammatory myofibroblastic tumors. It is likely that differential expression within the same IMT lesion explains disease resistance to some degree, as well.

Although the mainstay for successful treatment of IMT has been complete surgical excision, cases like that presented above often prove problematic for surgeon and oncologist, alike. Despite the difficulty, standard and novel pharmacotherapies, including NSAIDs, COX inhibitors, corticosteroids, and kinase inhibitors, are readily available for treatment of refractory, recurring, or nonresectable disease as evidenced in the literature on IMTs (Table 1). Variable reactivity to similar chemotherapeutic agents is common knowledge in IMT therapy. Nowhere is this more evident than in the previously presented case.

Recall that crizotinib, an ALK-1 inhibitor, was used by second referral center as therapy after unsuccessful surgical excision. This, no doubt, was initiated in hopes the scattered ALK positivity initially present in histologic sections would be inhibited, leading to regression and death of the tumor. The resistance of this particular lesion to such treatment, again, sheds light on the tremendous zonal and maturative expressivity profiles IMTs can possess. A second unique and fascinating characteristic of IMT is the often astounding regression when COX inhibitors like celecoxib and systemic steroids have been administered, a fact of which oncologists involved in the above case were well aware [14]. This highlights the vital role inflammatory mediators and immune dysregulation play in the survival and growth of these lesions [20, 33–35, 60, 61]. Surely the remarkable susceptibility shown in some IMTs when compared with the above resistant case should lead us to question the current standard of surgical excision with corticosteroid adjuvant therapy, especially when approaching a case that demonstrates all of the hallmarks of likely resistance and the potential for invasion. In such cases, sufficient evidence exists to support radiation therapy as first line adjuvant therapy. Indeed, most IMT cases involving successful treatment with radiation were of a resistant, refractory, or recurring nature [8].

In the age of detailed cytogenetic analysis, refined imaging techniques, and precisely targeted therapeutic regimens a greater degree of time should be dedicated to discovering a unique cytogenetic “profile” for each IMT case.

There is no denying this complex neoplasm demonstrates variation in the form of zonal expressivity, and overcoming this phenomenon will continue to be the challenge posed to all providers dealing with this rare tumor. Likewise, the role of specific cellular proteins such as ALK-1 will begin to serve as markers or the use of targeted therapy. Thus, physicians should emphasize effective determination of the cytogenetic profile of all IMTs, as well as a systematic and aggressive approach to IMTs presenting in areas shown to be refractory to many types of treatment, that is, paranasal sinuses. All things considered, the essential nature of taking a multidisciplinary approach with pathologist, surgeon, and medical and radiation oncologist providing concerted and comprehensive care is the foundation of proper IMT management.

## Figures and Tables

**Table 1:** Literature review: patients with maxillary sinus IMTs 1985 to 2014.

Age/sex	Presentation	MRI/CT reads	IHC stains	Treatment	Outcomes	Citation
22/F	Epistaxis and protrusion of left eye	Paranasal sinuses and L orbit expansion	POS: vimentin, SMA NEG: desmin, ALK-1	Resection, RT, CS, Chemo	Death	[32]

39/M	Nasal obstruction, supraorbital headaches	Vomer and ethmoid plate	NDA	Resection	24-month NED	[14]

16/F	L tinnitus, facial numbness, paresthesias	Sinus walls	NDA	(1) CS (2) CS	(1) Initial regression; recurrence 2 months later, (2) 2.5-year NED	[10]

39/M	L temporal headache, diplopia, paresthesias	Orbital floor	NDA	CS	1.5-year NED	[10]

27/F	R orbital swelling, trismus, diplopia	Infratemporal fossa, parapharyngeal space	POS: SMA	(1) Resection, (2) CS, (3) methotrexate	NDA	[11]

29/M	L facial numbness; swelling and pain in L maxillary sinus and upper teeth	L maxillary sinus and mild bony destruction	POS: vimentin, SMA, ALK-1 NEG: desmin, pancytokeratin, S100	NDA	NDA	[36]

38/M	Headache, R exopthalmia, R 6th nerve palsy	Invasion of right cavernous sinus, sphenoidal sinus	POS: SMA NEG: ALK-1	(1) CS, (2) RT (20 Gy)	2-year NED	[12]

NDA	NDA	NDA	8 cases: 7+ vimentin; 5+ SMA, desmin; 2+ S-100	6/8 partial and 1 complete maxillectomy; 1 no treatment	No recurrence in surgical patients	[37]

88/M	Nasal obstruction and foul smelling discharge	Nasal septum, infraorbital wall, L maxillary antrum	NEG: melanocytic and epithelial markers	Resection	9-month NED	[38]

2/F	Discomfort of R maxillary bone	NDA	NDA	Arterial embolization	5-year NED	[39]

24/M	Pain in L maxillary molars, swelling of L cheek, pulp necrosis of L 2nd molar	Lateral and superior L maxillary sinus walls	POS: SMA, b-catenin NEG: ALK-1, CD34	Resection	15-month NED	[40]

26/M	Diffuse facial pain and swelling, sensitivity in upper-right molar teeth	R medial wall and floor of maxillary sinus	POS: SMA, vimentin NEG: caldesmon, CD-68	(1) CS, (2) resection	(1) Regression, (2) 24-month NED	[41]

7/F	NDA	Expanding tumor without skull destruction	NDA	Resection	2-year NED	[42]

25/?	Pressure behind R eye, pain and swelling in R maxilla	NDA	NDA	Resection + CS (x2)	6-month NED	[43]

63/?	Pain and swelling of L face, numbness	L posterolateral wall	POS: vimentin NEG: SMA, S100	Resection, RT (50 Gy), Chemo → recurrence; RT (50 Gy)	Death	[44]

54/M	Swelling of L maxillary sinus and lower eyelid	Anterior maxillary sinus and infraorbital wall	POS: SMA, vimentin NEG: CD68, p53, S100	Resection → recurrence, resection	4-month NED	[45]

64/F	Nasal obstruction, epistaxis	Medial sinus wall remodeling	NDA	Resection	24-month NED	[46]

73/F	Vertigo, dysphagia, R retromolar swelling	No invasion	NDA	Incomplete resection + CS	Stable disease	[47]

6/F	Fever, painless swelling L cheek	Maxilla	NDA	CS	Partial regression	[48]

42/F	Nasal polyps	Orbital floor, lateral sinus wall	NDA	CS	Progression	[49]

41/M	Persistent necrotizing infections	Medial sinus wall	NDA	NDA	NDA	[50]

63/M	R facial pain, diplopia	Infraorbital wall, maxillary remodeling	NDA	NDA	NDA	[50]

67/M	Epistaxis	Ethmomaxillary plate	NDA	NDA	NDA	[50]

58/M	Epistaxis, L cheek swelling	Infraorbital wall	NDA	CS	1-month regression	[50]

15/M	R eye pain, R facial swelling, trismus	Orbital floor, medial wall	NDA	(1) CS + RT, (2) resection	(1) Stable disease, (2) NDA	[50]

48/M	L nasal obstruction	Orbital floors, sinus remodeling	NDA	NDA	NDA	[50]

15/M	R eye pain, R facial swelling, epistaxis	Invasion of medial/lateral sinus walls	NDA	CS	2-month minimal regression	[51]

32/F	Facial pain, R cheek fullness	Invasion of anterolateral sinus wall	NDA	Resection	1-month NED	[52]

13/F	NDA	Invasion of bone	NDA	CS + resection	33-month stable, residual disease	[53]

NDA	NDA	No invasion evident	NDA	CS	Stable disease	[54]

NDA	NDA	Sinus, orbit, anterior cranial fossa invasion	NDA	CS	Stable disease	[54]

NDA	NDA	Sinus, orbit, anterior cranial fossa invasion	NDA	CS	Stable disease	[54]

36/M	Obstruction, trismus	Lateral and posterior walls of nasopharynx	NDA	CS	7-month w/o symptoms, residual pain	[55]

18/M	Obstruction	Invasion into nasal septum and inferior turbinate	Polyclonal kappa and lambda light chains	(1) Resection → recurred, (2) RT (40 Gy)	27-month NED	[56]

40/M	NDA	Nasal cavity, ethmoid sinus	NDA	Resection	1.5-month stable, residual disease	[57]

83/M	NDA	Pterygomaxillary fossa	NDA	Resection	26-month NED	[58]

67/M	Dysphagia	Parapharyngeal mass	NDA	CS	4-year NED	[59]

63/F	R cheek swelling	Bone invasion of maxillary sinus	NDA	(1) RT (50 Gy), (2) CS, (3) cytoxan	Partial regression	[59]

55/M	Hypesthesia lower lip and jaw, progressive trismus	No bone or muscular invasion	NDA	Resection	1-year NED	[59]

NDA: no data available; RT: radiotherapy; NED: no evidence of disease; CS: corticosteroids.

## References

[1] Brunn H. (1939). Two interesting benign lung tumors of contradictory histopathology. *The Journal of Thoracic and Cardiovascular Surgery*.

[2] Umiker W. O., Iverson L. C. (1954). Post inflammatory tumor of the lung: report of four cases simulating xanthoma, fibroma or plasma cell granuloma. *The Journal of Thoracic Surgery*.

[3] Coffin C. M., Watterson J., Priest J. R., Dehner L. P. (1995). Extrapulmonary inflammatory myofibroblastic tumor (inflammatory pseudotumor): a clinicopathologic and immunohistochemical study of 84 cases. *The American Journal of Surgical Pathology*.

[4] Palaskar S., Koshti S., Maralingannavar M., Bartake A. (2011). Inflammatory myofibroblastic tumor. *Contemporary Clinical Dentistry*.

[5] Gale N., Zidar N., Podboj J., Volavšek M., Luzar B. (2003). Inflammatory myofibroblastic tumour of paranasal sinuses with fatal outcome: reactive lesion or tumour?. *Journal of Clinical Pathology*.

[6] Shek A. W. H., Wu P. C., Samman N. (1996). Inflammatory pseudotumour of the mouth and maxilla. *Journal of Clinical Pathology*.

[7] Ruaux C., Noret P., Godey B. (2001). Inflammatory pseudotumour of the nasal cavity and sinuses. *The Journal of Laryngology & Otology*.

[8] Newlin H. E., Werning J. W., Mendenhall W. M. (2005). Plasma cell granuloma of the maxillary sinus: a case report and literature review. *Head and Neck*.

[9] de Vuysere S., Hermans R., Sciot R., Crevits I., Marchal G. (1999). Extraorbital inflammatory pseudotumor of the head and neck: CT and MR findings in three patients. *American Journal of Neuroradiology*.

[10] Chong S., Teh C. S. L., Singh S., Seong M. K., Viswaraja S. (2014). Aggressive inflammatory pseudotumor of the maxillary sinus and orbit. *Ear, Nose & Throat Journal*.

[11] Salehinejad J., Pazouki M., Gerayeli M. A. (2013). Malignant inflammatory myofibroblastic tumor of the maxillary sinus. *Journal of Oral and Maxillofacial Pathology*.

[12] Yuan X. P., Li C. X., Cao Y., Singh S., Zhong R. (2012). Inflammatory myofibroblastic tumour of the maxillary sinus: CT and MRI findings. *Clinical Radiology*.

[13] Firat O., Ozturk S., Akalin T., Coker A. (2009). Inflammatory myofibroblastic tumour. *Canadian Journal of Surgery*.

[14] Chavez C., Hoffman M. A. (2013). Complete remission of ALK-negative plasma cell granuloma (inflammatory myofibroblastic tumor) of the lung induced by celecoxib: a case report and review of the literature. *Oncology Letters*.

[15] Butrynski J. E., D'Adamo D. R., Hornick J. L. (2010). Crizotinib in ALK-rearranged inflammatory myofibroblastic tumor. *New England Journal of Medicine*.

[16] Su L. D., Atayde-Perez A., Sheldon S., Fletcher J. A., Weiss S. W. (1998). Inflammatory myofibroblastic tumor: cytogenetic evidence supporting clonal origin. *Modern Pathology*.

[17] Lawrence B., Perez-Atayde A., Hibbard M. K. (2000). *TPM3-ALK* and *TPM4-ALK* oncogenes in inflammatory myofibroblastic tumors. *The American Journal of Pathology*.

[18] Coffin C. M., Patel A., Perkins S., Elenitoba-Johnson K. S. J., Perlman E., Griffin C. A. (2001). ALK1 and p80 expression and chromosomal rearrangements involving 2p23 in inflammatory myofibroblastic tumor. *Modern Pathology*.

[19] Dehner L. P. (2000). The enigmatic inflammatory pseudotumours: the current state of our understanding, or misunderstanding. *The Journal of Pathology*.

[20] Gómez-Román J. J., Ocejo-Vinyals G., Sánchez-Velasco P., Nieto E. H., Leyva-Cobián F., Val-Bernal J. F. (2000). Presence of human herpesvirus-8 DNA sequences and overexpression of human IL-6 and cyclin D1 in inflammatory myofibroblastic tumor (inflammatory pseudotumor). *Laboratory Investigation*.

[21] Pettinato G., Manivel J. C., de Rosa N., Dehner L. P. (1990). Inflammatory myofibroblastic tumor (plasma cell granuloma). Clinicopathologic study of 20 cases with immunohistochemical and ultrastructural observations. *The American Journal of Clinical Pathology*.

[22] Ramachandra S., Hollowood K., Bisceglia M., Fletcher C. D. M. (1995). Inflammatory pseudotumour of soft tissues: a clinicopathological and immunohistochemical analysis of 18 cases. *Histopathology*.

[23] Hussong J. W., Brown M., Perkins S. L. (1999). Comparison of DNA ploidy, histologic, and immunohistochemical findings with clinical outcome in inflammatory myofibroblastic tumors. *Modern Pathology*.

[24] Souid A. K., Ziemba M. C., Dubansky A. S. (1993). Inflammatory myofibroblastic tumor in children. *Cancer*.

[25] Cook J. R., Dehner L. P., Collins M. H. (2001). Anaplastic lymphoma kinase (ALK) expression in the inflammatory myofibroblastic tumor: a comparative immunohistochemical study. *The American Journal of Surgical Pathology*.

[26] Coffin C. M., Hornick J. L., Fletcher C. D. M. (2007). Inflammatory myofibroblastic tumor: comparison of clinicopathologic, histologic, and immunohistochemical features including ALK expression in atypical and aggressive cases. *The American Journal of Surgical Pathology*.

[27] Tang T. T., Segura A. D., Oechler H. W. (1990). Inflammatory myofibrohistiocytic proliferation simulating sarcoma in children. *Cancer*.

[28] Day D. L., Sane S., Dehner L. P. (1986). Inflammatory pseudotumor of the mesentery and small intestine. *Pediatric Radiology*.

[29] Meis J. M., Enzinger F. M. (1991). Inflammatory fibrosarcoma of the mesentery and retroperitoneum: a tumor closely simulating inflammatory pseudotumor. *American Journal of Surgical Pathology*.

[30] Myint M. A., Medeiros L. J., Sulaiman R. A., Aswad B. I., Glantz L. (1994). Inflammatory pseudotumor of the ileum. A report of a multifocal, transmural lesion with regional lymph node involvement. *Archives of Pathology and Laboratory Medicine*.

[31] Al-Sindi K. A., Al-Shehabi M. H., Al-Khalifa S. A. (2007). Inflammatory myofibroblastic tumor of paranasal sinuses. *Saudi Medical Journal*.

[32] Coffin C. M., Humphrey P. A., Dehner L. P. (1998). Extrapulmonary inflammatory myofibroblastic tumor: a clinical and pathological survey. *Seminars in Diagnostic Pathology*.

[33] Yoshizaki K., Matsuda T., Nishimoto N. (1989). Pathogenic significance of interleukin-6 (IL-6/BSF-2) in Castleman's disease. *Blood*.

[34] Rohrlich P., Peuchmaur M., De Napoli Cocci S. (1995). Interleukin-6 and interleukin-1*β* production in a pediatric plasma cell granuloma of the lung. *American Journal of Surgical Pathology*.

[35] Azuno Y., Yaga K., Suehiro Y., Ariyama S., Oga A. (2003). Inflammatory myoblastic tumor of the uterus and interleukin-6. *American Journal of Obstetrics and Gynecology*.

[36] Maire J.-P., Eimer S., San Galli F. (2013). Inflammatory myofibroblastic tumour of the skull base. *Case Reports in Otolaryngology*.

[37] Amin M., Ali R., Kennedy S., Timon C. (2012). Inflammatory myofibroblastic tumor of the nose and paranasal sinuses masquerading as a malignancy. *Ear, Nose and Throat Journal*.

[38] Murai A., Sugiu K., Kariya S., Nishizaki K. (2011). Transcatheter arterial embolisation for paediatric inflammatory pseudotumour of the maxillary sinus. *Journal of Laryngology and Otology*.

[39] Kim S.-Y., Yang S.-E. (2011). Inflammatory myofibroblastic tumor of the maxillary sinus related with pulp necrosis of maxillary teeth: case report. *Oral Surgery, Oral Medicine, Oral Pathology, Oral Radiology and Endodontology*.

[40] Naveen J., Sonalika W. G., Prabhu S., Gopalkrishnan K. (2011). Inflammatory pseudotumor of maxillary sinus: mimicking as an aggressive malignancy. *Journal of Oral and Maxillofacial Pathology*.

[41] Lawson S. L. A., Azoumah D. K., Lawson-Evi K. (2010). Imflammatory myofibroblastic tumour of nose and paranasal sinuses in a little girl of 7-year-old. *Archives de Pediatrie*.

[42] Kostka E., Guntinas-Lichius O., Wittekindt C. (2010). Unilateral recurrent tumor of the nasal cavitity and the paranasal sinuses. *Laryngo- Rhino- Otologie*.

[43] Zhou S.-H., Ruan L.-X., Xu Y.-Y., Wang S.-Q., Ren G.-P., Ling L. (2004). Inflammatory myofibroblastic tumour in the left maxillary sinus: a case report. *Chinese Medical Journal*.

[44] Karakök M., Özer E., Sar I. (2002). Inflammatory myofibroblastic tumor (inflammatory pseudotumor) of the maxillary sinus mimicking malignancy: a case report of an unusual location (is that a true neoplasm?). *Auris Nasus Larynx*.

[45] Lee H.-M., Choi G., Choi C. S., Kim C. H., Lee S. H. (2001). Inflammatory pseudotumor of the maxillary sinus. *Otolaryngology—Head and Neck Surgery*.

[46] Nakayama K., Inoue Y., Aiba T., Kono K., Wakasa K., Yamada R. (2001). Unusual CT and MR findings of inflammatory pseudotumor in the parapharyngeal space: case report. *American Journal of Neuroradiology*.

[47] de Oliveira Ribeiro A. C., Joshi V. M., Funkhouser W. K., Mukherji S. K. (2001). Inflammatory myofibroblastic tumor involving the pterygopalatine fossa. *The American Journal of Neuroradiology*.

[48] Batsakis J. G., Luna M. A., El-Naggar A. K., Goepfert H. (1995). `Inflammatory pseudotumor': what is it? How does it behave?. *Annals of Otology, Rhinology and Laryngology*.

[49] Som P. M., Brandwein M. S., Maldjian C., Reino A. J., Lawson W. (1994). Inflammatory pseudotumor of the maxillary sinus: CT and MR findings in six cases. *The American Journal of Roentgenology*.

[50] Maldjian J. A., Norton K. I., Groisman G. M., Som P. M. (1994). Inflammatory pseudotumor of the maxillary sinus in a 15-year-old boy. *The American Journal of Neuroradiology*.

[51] Muzaffar M., Hussain S. I., Chughtal A. (1994). Plasma cell granuloma: maxillary sinuses. *The Journal of Laryngology & Otology*.

[52] Foo T. H., Poh W. T. (1993). Fibro-inflammatory pseudotumour in the maxillary sinus. *Singapore Medical Journal*.

[53] Huang D. L. (1993). Inflammatory pseudotumor of nasal cavity. *Zhonghua Er Bi Yan Hou Ke Za Zhi*.

[54] Hytiroglou P., Brandwein M. S., Strauchen J. A., Mirante J. P., Urken M. L., Biller H. F. (1992). Inflammatory pseudotumor of the parapharyngeal space: case report and review of the literature. *Head and Neck*.

[55] Seider M. J., Cleary K. R., van Tassel P. (1991). Plasma cell granuloma of the nasal cavity treated by radiation therapy. *Cancer*.

[56] de Miguel García F., Bori Aiguabella M. A., Fernández Liesa R., Rivares Esteban J., Martínez-Berganza y Asensio R., Vicente González E. A. (1990). Inflammatory pseudotumor of the paranasal sinuses. *Acta Otorrinolaringologica Espanola*.

[57] Takimoto T., Kathoh T., Ohmura T., Kamide M., Nishimura T., Umeda R. (1990). Inflammatory pseudotumour of the maxillary sinus mimicking malignancy. *Rhinology*.

[58] Weisman R. A., Osguthorpe J. D. (1988). Pseudotumor of the head and neck masquerading as neoplasia. *Laryngoscope*.

[59] Keen M., Conley J., McBride T., Mutter G., Silver J. (1986). Pseudotumor of the pterygomaxillary space presenting as anesthesia of the mandibular nerve. *Laryngoscope*.

[60] Berger A., Kim C., Hagstrom N., Ferrer F. (2007). Successful preoperative treatment of pediatric bladder inflammatory myofibroblastic tumor with anti-inflammatory therapy. *Urology*.

[61] Germanidis G., Xanthakis I., Tsitouridis I. (2005). Regression of inflammatory myofibroblastic tumor of the gastrointestinal tract under infliximab treatment. *Digestive Diseases and Sciences*.

